# Dendritic Cells and Their Role in Cardiovascular Diseases: A View on Human Studies

**DOI:** 10.1155/2016/5946807

**Published:** 2016-03-20

**Authors:** Maja-Theresa Dieterlen, Katja John, Hermann Reichenspurner, Friedrich W. Mohr, Markus J. Barten

**Affiliations:** ^1^Department of Cardiac Surgery, Heart Center, Helios Clinic, University Hospital Leipzig, 04289 Leipzig, Germany; ^2^Department of Cardiovascular Surgery, University Heart Center Hamburg, 20246 Hamburg, Germany

## Abstract

The antigen-presenting dendritic cells (DCs) are key to the immunological response, with different functions ascribed ranging from cellular immune activation to induction of tolerance. Such immunological responses are involved in the pathophysiological mechanisms of cardiovascular diseases, with DCs shown to play a role in atherosclerosis, hypertension, and heart failure and most notably following heart transplantation. A better understanding of the interplay between the immune system and cardiovascular diseases will therefore be critical for developing novel therapeutic treatments as well as innovative monitoring tools for disease progression. As such, the present review will provide an overview of DCs involvement in the pathophysiology of cardiovascular diseases and how targeting these cells may have beneficial effects for the prognosis of patients.

## 1. Introduction

Dendritic cells (DCs) and their antigen-presenting properties possess a central role in the immune system, with many diseases associated with a heightened immune response. Among these, cardiovascular diseases (CVDs) represent the most frequent causes of death worldwide, with an estimated 17.3 million deaths per year [1]. While most research has been done in the field of heart transplantation, more recently, DCs were also shown to be involved in numerous other CVDs. This present review, therefore, will discuss the underlying role of DCs in the immunological mechanisms that underpin the development and progression of CVDs. In contrast to previous reviews, the present essay will focus exclusively on human rather than animal studies, as the different phenotypical and functional DC subsets between groups [2] can often lead to misleading conclusions.

## 2. Immunological Mechanisms

DCs patrol the blood and peripheral tissue to detect foreign and pathogenic antigens. According to their hematopoietic origin, DCs can be divided into myeloid DCs (mDCs) and plasmacytoid DCs (pDCs). Additionally, numerous surface markers have been shown to be useful for the identification of multiple DC subsets. For example, blood dendritic cell antigens (BDCA1–BDCA4) can be used to discriminate among DC types [3, 4], while further markers can also classify DCs such as S100 (expressed in mature and immature DCs), CD1a (expressed mainly in immature DCs), and CD83 (expressed mainly in mature DCs). Furthermore, specialized “cardiac” DCs have been found in the human heart [5–7], with immunohistochemical and morphological analyses revealing a subtype of DC expressing human leukocyte antigen- (HLA-) DR (but not S100, CD1a, CD21, CD23, and CD35 [5]). This different surface marker profile compared to ordinary DCs therefore led to the hypothesis that cardiac DCs change their characteristics depending on their location either in the blood vessels or in the heart [5].

The detection of foreign or pathogenic antigens (pathogen-associated molecular patterns, PAMPs) as well as tissue damage and inflammation (damage-associated molecular patterns, DAMPs) leads to phagocytosis of antigens by DCs, the latter then expressing the maturation marker CD83 and class I and class II major histocompatibility complexes (MHC). Mature, antigen-presenting DCs migrate to secondary lymphoid tissue where they present antigens to T cells [8]. While the homing of immature DCs is regulated by chemokine receptors (CCRs) on the DC surface, homing of mature DCs to regional lymph nodes is mediated by CCR7 and CXC-chemokine receptor type 4 (CXCR4) [9–11]. In the secondary lymphoid tissue, T cells are activated by receiving three signals from mature DCs as follows: (1) mature DCs present an antigen bound to a MHC molecule; (2) T cells then require costimulators such as CD80 or CD86; and (3) cytokines such as IL-12, IL-23, and IL-27 finally are secreted [12]. T cell activation leads to proliferation and differentiation of T cells into regulatory T cells (T_regs_), T helper cells (T_H_), or killer T cells. In general, DCs can activate all types of effector T cells and regulate activation and regulation of immune responses, which are both involved in disease patterns of CVDs.

## 3. DCs in Atherosclerosis and Aortocoronary Bypass

Atherosclerosis is the dominant cause of CVDs leading to myocardial infarction (MI), heart failure (HF), or stroke [13]. The investigation of the underlying pathophysiological mechanisms shows that immune cells such as T cells, monocytes, and DCs invade the vascular wall stimulated by oxidized LDL, TNF-*α*, and hypoxia [14, 15], which are often found in atherosclerotic lesions where they produce proinflammatory cytokines [16]. Both PAMPs and DAMPs can activate DCs [17] which subsequently mature, while further atherogenic factors in the vascular wall such as oxidized low-density lipoprotein (LDL) cholesterol [18], advanced glycation end products (AGE) [19], nicotine [20], insulin [21], and angiotensin II [22] also have the capacity to induce the maturation of DCs. Mature DCs activate T cells and initiate the upregulation of DC licensing factors such as CD40L [17]. These processes contribute to chronic vascular inflammation and form the basis for vascular obliteration.

Several reports on immunohistochemical analyses of carotid specimens raised the suggestion that DCs contribute to plaque destabilization, possibly through activation of T cells [23–26]. Yilmaz and colleagues analysed mDCs in atherosclerotic plaques in 44 carotid specimens and reported that advanced plaques had higher numbers of mDCs and a higher percentage of mature mDCs than initial lesions [23]. These observations were also confirmed in another patient study (*n* = 29), when unstable compared to stable plaques showed a 1.6-fold increase in both fascin^+^ mDCs and S100^+^ DCs, while a 5.9-fold increase of mature CD83^+^ mDCs was observed [26]. However, while these observations could not be detected in pDCs [26], alternative DC markers such as BDCA-1 and BDCA-2 have revealed that mDCs and pDCs are indeed recruited to advanced plaques [27].

In addition, the research work of the Weyand laboratory further showed that 53% of the 30 carotid endarterectomy samples contained CD123^+^ pDCs, but also CD11c^+^ DC-Sign^+^ fascin^+^ mDCs, which are both located either in the shoulder region of the plaque or at the plaque base. The mDC/pDC ratio in the plaques was 2.7, and further characterization of the pDCs revealed that these cells were the main source of interferon-*α*. The number of pDCs as well as interferon-*α* transcript concentrations strongly correlated with plaque instability in the tissue samples [24, 25]. Further research on the cytokine and chemokine expression in atherosclerotic plaques from coronary artery disease (CAD) patients revealed that the T cell cytokines, interferon-*γ* and TNF-*α*, as well as DC chemokines, CCL19 and CCL21, are increased in patients with ischemic symptoms compared to asymptomatic patients [28].

Beyond DC tissue analyses, circulating DCs hold significant value in patients suffering from atherosclerosis, as supported by CAD patients having increased number of DCs in the atherosclerotic vascular wall concomitant with decreased levels of circulating DCs in the blood [29–33]. While Yilmaz et al. reported a reduction of circulating mDCs, pDCs, and total DCs in patients with advanced CAD [31] and mDCs in patients with angina pectoris and MI [29], Van Vré et al. found that absolute and relative numbers of circulating pDCs were lowered in 18 CAD patients compared to age- and sex-matched controls [30]. Interestingly, the same group further reported an inverse correlation between mDCs and IL-6 and C-reactive protein, suggesting that these cytokines may be involved in their regulation [32]. Other factors may also play a role, such as IL-23 and IL-23R [34] and tyrosine kinase 3 ligand (Flt3L) [33], which have also been correlated to pDC levels.

A large clinical study provided further evidence of a strong association between the roles of DCs in CAD, with 290 patients classified as “early CAD,” “moderate CAD,” “advanced CAD,” and “CAD excluded” by coronary angiogram. In summary, the study demonstrated an inverse correlation between the CAD score and mDCs, pDCs, and total DCs, which were also independent predictors of CAD [31]. Yet noteworthy, patients undergoing percutaneous coronary intervention (PCI) or coronary artery bypass grafting (CABG) had lower total DCs and both DC subsets (mDCs and pDCs) compared to no intervention, suggesting that DC levels might be predictive of the targeted therapy after coronary angiogram [31].

Increased levels of DCs are also present in stenotic vein coronary bypass grafts [35]. In comparison to atherosclerosis of coronary arteries, the degeneration of vein grafts proceeds more rapidly [36] and finally leads to graft failure. In general, atherosclerosis develops in almost 50% of all vein grafts within ten years [37]. Cherian et al. investigated the presence of DCs in vein grafts and found DCs positive for S100 and CD1a in the vascular walls of these vessel grafts [35]. A further study on stenotic vein coronary bypass grafts demonstrated that DCs positive for the costimulatory molecule CD40 were clustered within the intima as well as in the media and adventitia [38]. In accordance with the results on human coronary bypass grafts, it has been demonstrated that T cells were accumulated in the vascular wall of saphenous vein grafts [39]. These data indicate that interactions between DCs and T cells are involved in the rapid development of atherosclerosis and degeneration of vein grafts, which finally promote eventual graft failure.

## 4. Hypertension and DCs

Hypertension is one of the most common chronic diseases, which promotes atherosclerosis and represents a major risk factor for CVD-related death [40]. A number of studies have suggested that immunological mechanisms, especially the inflammatory responses, are involved in hypertension [41–44]. Macrophages and lymphocytes infiltrate the interstitium in angiotensin II-induced hypertension [41], where T cells increase in the adventitia of blood vessels and secrete cytokines such as tumor necrosis factor-*α* (TNF-*α*) and IL-17 as well as NADPH oxidase [43, 45], which then lead to elevated blood pressure. This suggestion is reinforced by observations that immunosuppression causes reduced hypertension-induced end-organ damage while immunodeficiency reduces hypertension [42, 44].

While one research arm is related to the investigation of T cell variations in hypertension, another should represent T cell activating cells, specifically DCs. Indeed, Abbas et al. showed that hypertension activates DCs [34] and also further confirmed that reactive oxygen species (ROS) produced by DCs through phagocyte oxidase caused lipid oxidation, which resulted in accumulation of proteins that were oxidatively modified by highly reactive *γ*-ketoaldehydes (isoketals). The isoketal-modified proteins behave like DAMPs and activate DCs, which start to express IL-6, IL-1*β*, and IL-23 and the costimulators CD80 and CD86. The isoketal-pulsed DCs induced T cell proliferation, particularly of CD8^+^ and IFN-*γ* and IL-17A, with the latter shown to elevate blood pressure [46]. Thus, one key mechanism of hypertension could be related to an autoimmune component [47], which is supported by evidence that isoketal proteins were traceable in mDCs in hypertensive compared to normotensive controls. Unfortunately,* in vivo* studies of DCs in hypertension are not well investigated, with only one study on DCs in hypertensive patients [47].

## 5. DCs and Heart Failure

Inflammation and immune responses are processes that can lead to HF such as myocarditis and cardiomyopathy. Myocarditis is an inflammatory heart disease that can be initiated by infectious viruses (e.g., Coxsackie B virus, Parvovirus) or the parasite* Trypanosoma cruzi* [48]. These pathogens infect cardiomyocytes, which cause direct tissue injury but also initiate immune responses against pathogenic antigens that lead to further tissue damage. In addition, myocarditis has an autoimmune component driven by molecular “mimicry” between microbial and myocardial self-antigens [49]. Molecular mimicry means that specific structures of certain pathogens imitate defined cardiac self-antigens [50]. Subsequently, the T cell response against such microorganisms includes the expansion of self-reactive T cells with the potential to attack the myocardium [51]. For example, structural proteins from* Chlamydia* strains mimic myosin and induce myocarditis after immunizing mice with homologous* Chlamydia* peptides [52].

A histological study of cardiac samples from autopsied patients with myocarditis (*n* = 22) and from an age- und sex-matched control group (*n* = 20) provided evidence that HLA-DR-positive cardiac DCs proliferate in the acute phase of myocarditis [5]. Cardiac DCs showed typical morphology of DCs with large cellular processes and were in close contact with myocytes, suggesting that cardiac DCs exert a destructive effect on myocytes. This hypothesis is supported by the fact that necrotic lesions were surrounded by infiltrating HLA-DR-positive cells, with dendritic-forming mononuclear cells also in the immediate environment. Similarly, polymorphonuclear giant cells, cardiac DCs, and T cells have also been detected in active inflammatory lesions in chronic patients [5].

MI causes progressive remodeling of myocardial tissue and impairs contractile function, with eventual progression to HF [53]. Immunological and inflammatory processes play an important role in cardiac remodeling after MI [54], with DCs playing a central role in mediating immunological effects following MI by their role in the development of autoimmunological processes and maintenance of peripheral tolerance. For example, MI is characterized by the uptake and presentation of myocardial peptides by DCs resulting in T cell activation. The infiltration of mature activated CD11c^+^ CD11b^+^ DCs into the infarcted heart, as well as an association between mature DCs and the deterioration of left ventricle remodeling, has been demonstrated in experimental MI [7, 55]. Furthermore, DCs act as a potent immunoprotective regulator during the post-MI healing process via DC control of the monocyte/macrophage homeostasis [54], with it being demonstrated early after MI where DCs activate not only regulatory T cells (T_regs_), which are purported to prevent tissue-destructive autoimmunity after cardiac injury [56], but also other T cell subsets such as CD4^+^ T cells [56] and CD4^+^ T cells [57].

The effects on DC populations measured in human tissue are dependent on the type of MI [58, 59]. For example, a study on infarct tissue in patients with ST-elevation MI (STEMI), where patients with present or absent cardiac rupture were compared, found that CD209^+^ DCs and CD11^+^ DC infiltration was higher in patients with cardiac rupture, with a significant positive correlation between CD209^+^ DCs CD11c^+^ DCs and the extent of fibrosis further detected [58]. A more systematically designed study, where STEMI, non-STEMI (NSTEMI), and CAD (*n* = 123) patients were assessed for tissue-residing and circulating DCs [59], revealed circulating mDCs, pDCs, and total DCs decreased after acute MI, especially in STEMI patients, with higher DC numbers found in the infarcted myocardium. These results suggest that the lower numbers of circulating DCs after MI may be mediated by DC migration into the myocardium, which is indeed supported by several studies showing a reduction of circulating immature DCs [60], mDCs [61–63], or both, mDCs and pDCs [64] after MI. Further evidence shows that reduced circulating DC numbers return to baseline levels after seven days and do not change for a time period of three months later [64]. Moreover, the mDC/pDC ratio seems to be an additional important predictor to distinguish between coronary syndromes, as data has shown that a mDC/pDC ratio ≥ 4 allows patients suffering from acute coronary syndrome to be separated compared to those with stable angina pectoris or healthy controls [62].

In contrast, other studies have suggested that autoimmunological processes occur after MI in consequence to defective peripheral tolerance, as autoantibodies against myosin heavy chain, troponins, and *β*1-adrenoreceptors have been found in patients with dilative cardiomyopathy (DCM) or HF [65–69]. Ischemia induces changes not only to DCs but also in DCM, where data shows that chronic DC-driven myocardial inflammation results in ventricular functional impairment with hemodynamic characteristics resembling DCM [70]. Pistulli and coworkers investigated 72 endomyocardial biopsies from patients with diagnosed DCM and found a reduction in both myocardial DCs of all subtypes (mDCs, pDCs, mature DCs, and immature DCs) and maturation markers (fascin, CD11c, CD209, CD83, and CD304), as well as an inverse correlation of DCs with tissue fibrosis. Furthermore, a reduction of mDCs in DCM hearts in concert with positive testing for cardiotropic viruses has been reported, which raises the hypothesis about a connection between mDCs and myocardial virus clearance [71].

The situation of DCs looks different in whole blood of patients with chronic HF (CHF) [72], where elevated mDC and mature DCs levels have been reported while pDCs were unchanged. It has been hypothesized that the shift of the mDC/pDC balance towards mature mDCs may be associated with T_H_1 biased immune responses in later stages of CHF [72]. A few years after this study was published, Athanassopoulos et al. reported that patients with end-stage CHF of NYHA category III and category IV had comparable levels of circulating DC subsets to NYHA II patients and healthy volunteers [73]. In contrast, Sugi et al. showed that patients with NYHA III and NYHA IV had lower counts of circulating mDCs and pDCs [74]. After treatment of decompensated HF with optimized oral and intravenous heart insufficiency medication, the reduction of circulating mDCs and pDCs was restored and increased during the following weeks. Overall, these findings suggest that the role of DCs in the pathophysiology of HF is controversial, with further studies required to clarify the associations between DCs and disease development.

## 6. DCs and Heart Transplantation

**Figure 1:**
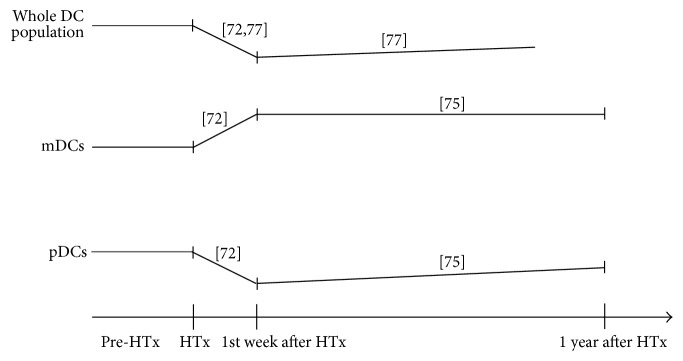
Changes of dendritic cells and their subsets following heart transplantation. Increases and decreases of the whole DC population and the subsets mDCs and pDCs following heart transplantation were visualized. The appropriate references were added in squared brackets. DCs: dendritic cells; HTx: heart transplantation; mDCs: myeloid dendritic cells; pDCs: plasmacytoid dendritic cells.

The central role of DCs in mediating inflammation and immune tolerance has been demonstrated for MI, myocarditis, DCM, and HF. Aside from these diseases, DCs hold a key role in the immunological processes that are connected to allograft rejection. Inflammatory processes and allograft rejection belong to the major complications after heart transplantation (HTx), whereas immunological tolerance is closely related to a positive outcome following HTx. Clinical studies have focused on monitoring DCs after HTx, with the aim of investigating whether DCs are a valuable marker of immune function status after transplantation [75]. Athanassopoulos et al. examined total peripheral blood mDC and pDC subsets expressing CD83 and CCR7 in 16 patients before HTx and one week after HTx compared to 14 healthy controls [72]. A further study of this group investigated DCs and their subsets up to 38 weeks after HTx in 20 HTx patients [76]. Dieterlen et al. investigated DCs and mDC and pDC subsets in the first twelve months after HTx in 46 HTx patients [75]. In summary, these studies revealed that patients had higher percentage of DCs before transplantation (Figure 1). Within the first week after HTx, a marked decrease in both the percentage of DCs and that of pDCs was observed but an increase in the percentage of mDCs was observed [72, 76], with the number of the whole DC populations increasing continuously during the following months [76]. A more detailed analysis of DC subsets showed that pDCs increased during the first year after HTx, while mDCs remained constant within that time [75]. In summary, these studies demonstrated that DC incidence and subset distribution differed substantially between recipients before and after HTx as well as in healthy subjects. Thus, it has been stated that DC homeostasis is altered after transplantation (see Figure 1) [77].

Surgery and stress cause a transient increase of peripheral blood DCs [78, 79], and therefore the decrease of DCs and their subsets in the early posttransplantation period has to be ascribed to the immunosuppressive treatment by antithymocyte globulin, corticosteroids, calcineurin inhibitors, or mycophenolate mofetil [72, 76]. The different effects and mechanisms of immunosuppressive drugs on DCs are shown in Table 1.

Immunosuppressive drugs keep DC subsets in an immature state, where there is a potent effect not only on maturation but also on the migration characteristics of mDCs and pDCs [90–92]. These findings are in accordance with a report on circulating DCs, which found lack in the expression of the maturation markers CD83 and CCR7 early after HTx [72]. Furthermore, it has been reported that mycophenolate mofetil influenced phenotype and function during the maturation process, and cyclosporine A and tacrolimus inhibited DC migration [93]. A direct comparison of cyclosporine A- (*n* = 14) and tacrolimus-treated (*n* = 14) HTx recipients showed that the percentage of mDC values was higher and percentage of pDC values was lower in the cyclosporine A-treated group than in the tacrolimus-treated group. Additionally, monitoring the same study cohort for DC subsets over a period of six months showed that mDC values only differed at study onset and aligned up to month 6. In contrast, pDC values and the pDC/mDC ratio differed significantly at all study time points (day 0, month 3, and month 6) [94]. Barten et al. monitored DCs in 16 HTx patients with regard to the immunosuppressive regimen after conversion of calcineurin inhibitors or sirolimus to everolimus [95]. Regardless of the immunosuppressive regimen, HTx patients had higher percentage of mDCs compared to healthy controls, whereas pDCs were only significantly lower in patients with conversion from calcineurin inhibitors to everolimus. Sirolimus maintenance therapy caused a similar percentage of pDCs compared to controls, with a shift to pDCs in the pDCs/mDCs ratio compared to recipients with calcineurin inhibitor therapy. Furthermore, an additional elevated shift in the pDC/mDC ratio towards pDCs after conversion from calcineurin inhibitors or sirolimus to everolimus has been observed, which was comparably higher than controls [95].

As mentioned above, DC monitoring is performed to investigate DCs as a marker of immune function status. Thus, studies often correlate DC numbers and acute cellular rejection (ACR). Two different study cohorts have been investigated regarding the influence of rejection episodes on DCs. Firstly, John et al. analysed twenty-eight HTx patients and found that the percentage of pDCs was lower in HTx recipients with rejection compared to HTx recipients without ACR. In contrast, no differences between rejector and nonrejectors have been detected for mDCs [94]. Secondly, a study cohort including twenty-one HTx patients correlated DC subsets with different rejection grades [76, 77]. A negative association of mDCs but not of pDCs with the rejection grade determined from endomyocardial biopsies has been found. The number of peripheral blood DCs and the mDC/pDC ratio decreased markedly during ACR episodes, and a lower mDC number has been documented even three months after ACR [77]. Furthermore, Athanassopoulos et al. showed that aberrant DC reconstitution is related to adverse clinical outcome after HTx [76].

The central role of DCs in immunological processes led to the development of cellular vaccination strategies aiming to induce transplant tolerance [96]. DCs that are involved in processes leading to tolerance were named “tolerogenic DCs” (tolDCs). tolDCs have immunosuppressive characteristics and exert their function via passive (lack of costimulatory signals) and active (presence of inhibitory signals) tolerance [97]. According to their organism of origin, tolDCs can be classified into “donor-derived tolDCs” and “recipient-derived tolDCs.” Furthermore, it is possible to generate tolDCs* in vitro*, for example, from monocytes [98], or to induce tolDCs* in vivo*.

One possibility for the* in vivo* induction of tolDCs is extracorporeal photopheresis (ECP) [99]. ECP is an apheresis technique that collects a portion of patients' venous whole blood in a medical device located outside the patients' body (extracorporeal). After separating the blood into its components by centrifugation, the fraction containing white blood cells is treated with the photosensitizing drug methoxsalen and UV-A light and then returned into the patients' circulation. The investigation of DC subsets in HTx patients (*n* = 25) during and after ECP treatment showed that almost 80% of the treated HTx patients had increased pDCs and regulatory T cells (T_regs_) [100]. The authors proposed classification criteria based on the individual courses of pDCs and T_regs_ to discriminate between patient specific responses to ECP therapy.

tolDCs induce tolerogenic immune reactions and immunomodulation faster and more frequently than immature DCs [97]. The mechanisms that are involved in immunomodulation are the IL-10- and TGF-*β*-driven differentiation of T_regs_, the cytokine production that promotes tolDC biology, and the cytokine expression of inhibitory molecules that regulate T cell responses [97]. Animal models demonstrated that cardiac allograft survival is prolonged by infusion of tolerogenic pDCs in combination with anti-CD40L therapy [101, 102]. At present, no clinical application of tolDCs is approved or under investigation for HTx. The ONE Study, which is an ongoing multicenter, prospective, and randomized clinical trial, is the first study that evaluates immunomodulatory cellular therapy of ECP on tolDCs in kidney transplantation [103].

Irrespectively of the* in vitro* generation of tolDCs, different types of tolDCs have been found in humans* in vivo* [97]. DC-10 cells, a type of mDC expressing IL-10, have been identified by Gregori et al. [104]. This type of tolDC expressed inhibitory molecules (ILT-2, ILT-3, and ILT-4) and the costimulatory surface molecules CD40 and CD86 and trigger tolerogenic effects [104]. Natural tolerogenic pDCs differed in their tolerance-inducing properties compared to tolerogenic mDCs. These differences are caused by the biology of pDCs, which includes a less effective antigen presentation, different maturation characteristics, and expression of costimulatory molecules [105].

## 7. Conclusions

We have reviewed the role and alterations of DCs in CVD and also the current state-of-the-art research. While there remain numerous gaps and contrary findings related to the effects of DCs in different CVDs, many observations of human studies are based on circulating measurements despite distinct DCs residing in the tissue that are not detected in analyses of peripheral blood samples. Therefore, analyses of circulating DCs fail to provide information about the processes that are initiated after DC activation or tissue-specific DCs. Such limitations can be closed with further intensive preclinical and clinical research, which should include studies measuring circulating and tissue-residing DCs simultaneously. An interesting aspect, which is yet to be studied in prospective clinical trials, is the role of circulating DCs as immunological markers for CVDs.

While circulating DCs can be analysed by flow cytometry, tissue-residing DCs are measurable by immunohistochemistry or slide-based cytometry [106]. However, it has to be noted that the investigation of tissue-residing DCs requires biopsy material, which may pose additional risks for patients dependent on CVDs. While it is clinical routine to perform endomyocardial biopsies to detect graft rejection after HTx, this is not the case for patients with MI and atherosclerosis.

At present, only few human studies with low patient numbers compared DCs in the tissue with peripheral blood DCs (Table 2). In particular, the role of DCs in hypertension and in diseases leading to HF is still widely unexplored. Furthermore, many studies present conflicting results, which may be related to the different markers used for DC classification, as some study groups favor the classification via the expression of CD11 and others define DC subsets by the markers BDCA1–BDCA4, DC-1a, or S100. Thus, a consensus is urgently required on the functional and phenotypical DC classification in order to allow results to become more comparable.

Furthermore, the number of circulating DCs is extremely small with less than 1% of the leukocytes being DC subsets. Therefore, by using detection methods that are designed to identify very rare cell populations, such as flow cytometry, for example, this problem could be dramatically reduced. However, the establishment of high-throughput methods for clinical diagnostics of DCs is hindered for rare cell populations.

In conclusion, while DCs represent a cell type capable of modulating immunological processes in CVDs, only clinical studies investigating both the circulating and tissue-residing DCs will help further clarify the underlying mechanisms of how these cells exert their immunological effects in humans.

## Figures and Tables

**Table 1:** Effects of immunosuppressive drugs on dendritic cells.

Immunosuppressive drug	Effect on DCs	Reference
Anti-thymocyte globulin	(i) Binding of immature and mature DC subsets with the following induction of complement-mediated DC lysis	Monti et al., 2003 [80]

Cyclosporine A	(i) Interference with DC recirculation through cyclooxygenase-2 inhibition or prostaglandin E2 uncoupling with CCR7	Luft et al., 2002 [81]; Scandella et al., 2004 [82]
	(ii) Inhibition of DC migration by competitive inhibition of the lipid transporters MDR1 and MRP1	Randolph, 2001 [83]
	(iii) Inhibition of NF*κ*B production in DCs	Szabo et al., 2001 [84]

Sirolimus	(i) Inhibition of mDCs' IL-12 signaling	Chiang et al., 2004 [85]

Tacrolimus	(i) Suppression of DC allocostimulatory capacity by decreasing TNF-α and IL-12 secretion	Lagaraine and Lebranchu, 2003 [86]
	(ii) Inhibition of NF*κ*B production in DCs	Szabo et al., 2001 [84]

Mycophenolate mofetil	(i) Suppression of DC allocostimulatory capacity by decreasing TNF-α and IL-12 secretion	Lagaraine and Lebranchu, 2003 [86]

Prednisone/Dexamethasone	(i) Reduction of circulating pDC numbers	Moser et al., 1995 [87]
	(ii) Induction of pDC apoptosis	Boor et al., 2006 [88]
	(iii) Inhibition of DC function *in vivo*	Shodell and Siegal, 2001 [89]
	(iv) Inhibition of DC migration by competitive inhibition of the lipid transporters MDR1 and MRP1	Randolph, 2001 [83]

CCR7: chemokine receptor 7; DC/DCs: dendritic cell/dendritic cells; IL-12: interleukin-12; MDR1: multidrug resistance 1; MRP1: multidrug resistance protein; NF*κ*B: nuclear factor “kappa-light-chain-enhancer” of activated B-cells; pDC: plasmacytoid dendritic cell; TNF-α: tumor necrosis factor-α.

**Table 2:** Clinical studies on the role of dendritic cells in cardiovascular diseases.

Study	Type of cardiovascular disease	Study population	Major findings
	*Atherosclerosis*		

Cherian et al., 2000 [107]	Atherosclerosis	Patients with aortocoronary bypass (*n* = 12)	(i) DCs present in stenotic aortocoronary saphenous vein bypass grafts

Cherian et al., 2001 [35]	Atherosclerosis	Patients with aortocoronary bypass (*n* = 14) and healthy controls (*n* = 10)	(i) CD1a^+^/S100^+^ DCs present in stenotic saphenous vein bypass grafts but not in normal saphenous veins

Ozmen et al., 2001 [38]	Atherosclerosis	Patients with stenotic aortocoronary saphenous vein grafts (*n* = 3) and control carotid arteries (*n* = 8)	(i) CD40^+^ cells detected in stenotic grafts and carotid plaques (ii) CD40^+^/S100^+^ cells clustered within the intima, the media, and the adventitia

Yilmaz et al., 2006 [29]	Atherosclerosis	Patients with carotid endarterectomy (*n* = 44)	(i) Lower DC numbers in initial lesions than in advanced plaques (ii) DC number higher in stable than in vulnerable plaques (iii) 70% of DCs in advanced plaques with mature phenotype indicate functional activity of DCs

Van Vré et al., 2006 [30]	Atherosclerosis	CAD patients (*n* = 18) and controls (*n* = 18)	(i) Lower numbers and percentage of pDCs and mDCs in patients with CAD than in controls

Niessner et al., 2006 and 2007 [24, 25]	Atherosclerosis	Patients with carotid endarterectomy (*n* = 30)	(i) 53% of carotid samples with CD123^+^ pDCs and with CD11c^+^ DC-Sign^+^ fascin^+^ mDCs (ii) DCs localized in the shoulder region and at the base of the plaque (iii) pDCs are localized in the shoulder region and produce IFN-*α* (iv) IFN-*α* transcript concentrations correlated with plaque instability (v) mDC : pDC ratio of 2.7 in the plaques

Erbel et al., 2007 [28]	Atherosclerosis	Patients with carotid artery plaques (*n* = 57)	(i) Plaques from patients with ischemic complications with elevated levels of CD83, CCL19, and CCL21 (ii) Presence of CD83^+^ DCs in the shoulder region of unstable plaques

Yilmaz et al., 2009 [31]	Atherosclerosis	CAD patients (*n* = 290)	(i) Reduction of pDCs, mDCs, and DCs in advanced CAD patients (ii) Reduction of pDCs, mDCs, and DCs in patients with required percutaneous coronary intervention or coronary artery bypass grafting

Van Vré et al., 2010 [32]	Atherosclerosis	CAD patients (*n* = 46) and controls (*n* = 15)	(i) Decrease of total blood DCs, mDCs, and pDCs in CAD patients compared to controls (ii) Inverse association of IL-6 and hs-CRP with mDCs

Van Vré et al., 2011 [27]	Atherosclerosis	Patients with carotid endarterectomy (*n* = 22) or autopsy (*n* = 87)	(i) Accumulation of BDCA-1 and BDCA-2 near microvessels (ii) S100^+^ and fascin^+^ DCs increased from intimal thickening via pathological thickening, fibrous cap atheroma to complicated plaques

Van Brussel et al., 2011 [33]	Atherosclerosis	CAD patients (*n* = 15) and controls (*n* = 12)	(i) Circulating mDCs and pDCs declined in CAD patients (ii) Frequencies of CD86^+^ and CCR7^+^ mDCs, but not pDCs, declined in CAD patients (iii) Plasma Flt3L positively correlated with blood DC counts

Abbas et al., 2015 [34]	Atherosclerosis	Patients with carotid atherosclerosis (*n* = 177) and healthy controls (*n* = 24)	(i) pDCs with increased mRNA levels of IL-23 and IL-23R in atherosclerosis

Rohm et al., 2015 [26]	Atherosclerosis	Patients with carotid endarterectomy (*n* = 29)	(i) Higher numbers of fascin^+^, S100^+^, or CD83^+^ mDCs are unstable compared with stable plaques (ii) No differences between stable and unstable plaques for pDCs

	*Hypertension*		

Kirabo et al., 2014 [47]	Hypertension	Hypertensive patients (*n* = 142) and normotensive controls (*n* = 24)	(i) Elevated levels of isoketal-modified proteins in circulating monocytes and DCs in patients with hypertension (ii) Hypertension activates DCs, in large part by promoting the formation of isoketals

	*Heart failure associated diseases*		

Yokoyama et al., 2000 [5]	Myocarditis	Acute myocarditis patients (*n* = 22) and patients that died from noncardiac disease (*n* = 20)	(i) Cardiac DCs increase in the acute phase of myocarditis (ii) Cardiac DCs with long, slender dendritic processes and positive for HLA-DR, but negative for CD68

Athanassopoulos et al., 2004 [72]	HF, transplantation	HF/HTx patients (*n* = 16) and healthy controls (*n* = 14)	(i) Increase of blood DCs and mDCs in CHF patients (ii) Increase of mature DC subsets compared to controls

Yilmaz et al., 2006 [29]	MI	Angina pectoris (*n* = 39) and MI (*n* = 17) patients	(i) Reduced circulating mDCs in patients with angina pectoris and acute myocardial infarction compared to controls (ii) mDCs inversely correlated with C-reactive protein or IL-6 (iii) More mDC precursors in vulnerable carotid plaques than in stable ones

Athanassopoulos et al., 2009 [73]	HF	NYHA II patients (*n* = 12), NYHA III/IV patients (*n* = 28), and healthy controls (*n* = 18)	(i) NYHA III/IV patients with comparable percentage of circulating DC subsets (ii) Within NYHA III/IV patients: total DC levels in patients with nonischemic DCM higher than in patients with CAD, HF, and HCM (iii) Mature mDCs, but not pDCs, in DCM patients compared to patients with CAD, HCM, or other cardiac pathophysiologies

Sugi et al., 2011 [74]	HF	Patients with decompensated HF (*n* = 27)	(i) Circulating DC subsets lower in decompensated HF patients compared to controls (ii) HF treatment restored reduction and activation of circulating mDCs and pDCs (iii) Numbers of circulating DCs correlated with decreases of BNP and troponin-T (iv) Poor recovery of circulating DC numbers predictive of recurrence of decompensated HF

Kofler et al., 2011 [60]	MI	STEMI patients (*n* = 35), NSTEMI patients (*n* = 30), stable CAD patients (*n* = 15), and controls (*n* = 15)	(i) Downregulation of immature (CD1a^+^) DCs in STEMI, NSTEMI, and CAD patients (ii) Upregulation of mature (CD86^+^) DCs in CAD patients

Fukui et al., 2012 [64]	MI	AMI patients (*n* = 26), SAP patients (*n* = 19), and controls (*n* = 19)	(i) Circulating mDCs and pDCs lower in AMI group than in SAP or control group (ii) Numbers of circulating mDCs and pDCs returned to control levels 7 days after AMI and were stable until the next 3 months (iii) % CD40^+^ and CD83^+^ mDCs higher in AMI patients than in SAP group or controls (iv) % CD40^+^ and CD83^+^ pDCs were similar between the three groups

Carvalheiro et al., 2012 [63]	MI	AMI patients (*n* = 12) and healthy controls (*n* = 12)	(i) Lower frequency of circulating mDCs

Kretzschmar et al., 2012 [59]	MI	STEMI patients (*n* = 34), NSTEMI patients (*n* = 44), and controls (*n* = 45)	(i) Decrease of circulating mDCPs, pDCPs, and tDCPs in AMI patients with pronounced reduction in STEMI patients (ii) Higher DC number in infarcted myocardium than in control

Wen et al., 2013 [61]	MI	AMI patients (*n* = 50), SAP patients (*n* = 30), UAP patients (*n* = 56), and controls (*n* = 29)	(i) % circulating mDC precursors reduced in AMI and UAP patients compared to SAP patients and controls (ii) % circulating pDC precursors not different between the groups (iii) % circulating mDC precursors negatively correlated with severity and extent of coronary artery lesions

Pistulli et al., 2013 [71]	DCM	DCM patients (*n* = 72)	(i) Myocardial DCs of all subtypes and maturation stages decreased in DCM compared to controls (ii) T_regs_, apoptosis, and CCR7 overexpressed in DCM (iii) mDCs reduced in virus-positive endomyocardial biopsies (iv) mDC number correlated with positive change in EF at follow-up

Nagai et al., 2014 [58]	MI	STEMI patients with present (*n* = 13) or absent (*n* = 11) cardiac rupture	(i) CD209^+^ DC and CD11c^+^ DC infiltration increased in the rupture group (ii) Positive correlation between the number of infiltrating CD209^+^ DCs and CD11c^+^ DCs and the extent of reparative fibrosis

	*Transplantation*		

Athanassopoulos et al., 2004 [72]	Transplantation, HF	HTx patients (*n* = 16) and healthy controls (*n* = 14)	(i) Decrease of total DCs, mDCs, and pDCs one week after HTx (ii) % of circulating mDCs higher after HTx compared to CHF patients and controls (iii) Maturation status of DC subsets comparable to controls (but not the CCR7^+^ pDCs)

Athanassopoulos et al., 2005 [76]	Transplantation	HTx patients (*n* = 21)	(i) Reduced DC numbers up to week 38 after HTx (ii) Negative association of mDCs with rejection grade (iii) mDCs and their mature states decreased during AR episodes and are lower in rejectors than in nonrejectors

Athanassopoulos et al., 2005 [77]	Transplantation	HTx patients (*n* = 20 venous blood analyses; *n* = 14 EMB analyses)	(i) Total DC numbers decreased at the first week after HTx and remained lower than the pre-HTx condition until week 38 (ii) Negative association between mDCs, but not pDCs, and the diagnosed ISHLT rejection grade for the follow-up period

Barten et al., 2006 [95]	Transplantation	HTx patients (*n* = 16) and healthy controls (*n* = 20)	(i) Higher % of mDCs in HTx patients compared to controls (ii) % of pDCs were different in patients with conversion from calcineurin inhibitors to everolimus compared to healthy controls (iii) Mature mDCs did not differ between HTx patients and controls

John et al., 2014 [94]	Transplantation	HTx patients (*n* = 28)	(i) mDCs higher and pDCs lower in cyclosporine A-treated patients than in tacrolimus-treated patients (ii) pDC/mDC ratio higher at day 0, month 3, and month 6 in tacrolimus-treated patients than in cyclosporine A-treated patients

Dieterlen et al., 2015 [75]	Transplantation	HTx patients (*n* = 46)	(i) Increase of pDCs, but not for mDCs, in the first year after HTx (ii) No significant changes of the pDC/mDC ratio in the first year after HTx

AMI: acute myocardial infarction; AR: acute rejection; BDCA-1/BDCA-2/BDCA-3/BDCA-4: blood dendritic cell antigen-1/antigen-2/antigen-3/antigen-4; BNP: B-type natriuretic peptide; CAD: coronary artery disease; CD1a/CD11c/CD40/CD68/CD83/CD123/CD209: Cluster of Differentiation 1a/11c/40/68/83/123/209; CHF: chronic heart failure; CCL19/CCL21: chemokine ligands 19/21; DCM: dilative cardiomyopathy; DC/DCs: dendritic cell/dendritic cells; EF: ejection fraction; EMB: endomyocardial biopsy; Flt3L: FMS-like tyrosine kinase 3 ligand; HCM: hypertrophic cardiomyopathy; HF: heart failure; HLA-DR: human leukocyte antigen DR; hs-CRP: high sensitivity C-reactive protein; HTx: heart transplantation; IFN-*α*: interferon-*α*; IL-6/IL-23/IL-23R: interleukin 6/interleukin 23/interleukin 23-receptor; ISHLT: International Society of Heart and Lung Transplantation; mDCs: myeloid dendritic cells; mDCPs: myeloid dendritic cell precursors; MI: myocardial infarction; NSTEMI: non-ST-elevation myocardial infarction; NYHA II/III/IV: New York Heart Association grade II/III/IV; pDCs: plasmacytoid dendritic cells; pDCPs: plasmacytoid dendritic cell precursors; SAP: stable angina pectoris; STEMI: ST-elevation myocardial infarction; S100: calcium-binding protein with low molecular weight, marker for DCs; tDCPs: total dendritic cell precursors; T_regs_: regulatory T cells; UAP: unstable angina pectoris.
